# Les traumatismes abdominaux en Haïti

**DOI:** 10.5588/pha.23.0008

**Published:** 2023-08-01

**Authors:** S. J. Baptiste, W. van den Boogaard, J-P. Letoquart, J-G. NDong, G. Jonacé, L-F. Télémaque

**Affiliations:** 1 Centre Traumatologie Tabarre, Médecins Sans Frontières (MSF), Port-au-Prince, Haïti; 2 Département Médicale, Unité recherche opérationnel (LuxOR), MSF, Luxembourg; 3 Département Médical, MSF, Paris, France; 4 Département Chirurgie-traumatologie, Ministère de la Santé Publique et de la Population, Port-au-Prince, Haïti

**Keywords:** Caraïbe, devenir défavorable, politique traumatisme, violence

## Abstract

**CONTEXTE:**

Les traumatismes abdominaux (TA) semblent fréquents en Haïti confronté à des violences socio-politiques récurrents.

**OBJECTIF:**

Etudier les patients admis pour TA au centre traumatologie Médecins Sans Frontières (MSF) Tabarre (Port-au-Prince), et les circonstances de survenue.

**TYPE D’ETUDE:**

Ceci est une étude transversale sur des données rétrospectives de Janvier 2020 à Décembre 2021.

**RÉSULTATS:**

Sur 3 211 patients admis pour traumatisme, 541 (17,3%) avaient un TA, dont 500 (91,4%) en lien avec des événements socio-politiques. Leur âge médian était de 30 ans (intervalle interquartile [IQR] 23–38) ; 429 (85,8%) étaient masculin. Une blessure par balle était notée chez 371 (74,2%). La distance médiane entre le lieu de violence et l’hôpital était de 11 km (IQR 7–15) ; cependant, 9 (1,8%) étaient venus dans l’heure après le traumatisme ; la transfusion était non faite ou insuffisante chez 169 (33,8%). Une issue défavorable (décès, référence, sortie contre-avis médical) était notée chez 57 (11,4%), avec 8,0% de décès. L’instabilité politique était la principale cause de violence. Une issue défavorable était associée à une transfusion insuffisante (rapport de risque [RR] 2,4 ; IC 95% 1,4–4,3 ; *P = *0,006) ou à une blessure par balle (RR 2,4 ; IC 95% 1,1–5,2 ; *P = *0,002).

**CONCLUSION:**

Les TA par balle étaient fréquents durant la période des évènements socio-politiques 2020–2021. Le manque de produits sanguins a eu un impact négatif sur l’issue des patients. Les mesures de sécurité et la collecte de sang doivent toujours être maintenues et renforcées.

## Abstract (en)

**CONTEXT:**

Abdominal trauma (AT) appears to be frequent in Haiti, which is confronted with recurrent socio-political violence.

**OBJECTIVE:**

To study patients admitted for AT to the Médecins Sans Frontières (MSF) Tabarre trauma centre (Port-au-Prince), and the circumstances of occurrence.

**DESIGN:**

This was a cross-sectional study with retrospective data from January 2020 to December 2021.

**RESULTS:**

Of 3,211 patients admitted for trauma, 541 (17.3%) had an AT, of which 500 (91.4%) were related to sociopolitical events. Their median age was 30 years (interquartile range [IQR] 23–38); 429 (85.8%) were male. A gunshot wound was noted in 371 (74.2%). The median distance from the scene of violence to the hospital was 11 km (IQR 7–15); however, 9 (1.8%) came within 1 hour of the trauma; transfusion was not done or insufficient in 169 (33.8%). An adverse outcome (death, referral, discharge against medical advice) was noted in 57 (11.4%), with 8.0% of deaths. Political instability was the main cause of violence. An adverse outcome was associated with inadequate transfusion (hazard ratio [HR] 2.4, 95% CI 1.4–4.3; *P* = 0.006) or gunshot wound (HR 2.4, 95% CI 1.1–5.2; *P* = 0.002).

**CONCLUSION:**

Firearm injuries were common during the period of sociopolitical events 2020–2021. The lack of blood products had a negative impact on patient outcomes. Safety measures and blood collection still need to be maintained and strengthened.

Les traumatismes représentent l’une des causes majeures de morbi-mortalité dans le monde, responsables de 4,4 millions de décès chaque année ;^1,2^ 12% de ces traumatismes seraient directement liés aux homicides ou aux affrontements entre groupes armés.^3^ Parmi eux, les traumatismes abdominaux (TA) font partie des urgences les plus fréquentes, représentant 15–20% des traumatismes, avec une mortalité avoisinant 20%.^4^ La situation est particulièrement préoccupante dans les pays à revenus faibles ou intermédiaires, où la prévalence peut atteindre 55% des traumatismes, justifiée entre autres par l’ampleur des violences et la fréquence des agressions par armes souvent liées à des événements socio-politiques, comme démontré dans des pays comme l’Afrique du Sud, le Nigeria,^5,6^ ou encore en Amérique latine dans les Caraïbes.^7^

Dans la littérature, les causes, les types de lésions, la sévérité, les facteurs pronostiques sont de contexte-spécifiques. Ainsi, dans un travail mené au Nigéria, avec une mortalité de 5,6%, le choc hypovolémique, le clivage entre les milieux urbain et rural étaient parmi les facteurs pronostiques défavorables.^5^ Dans un autre en Afrique du Sud, où la mortalité était de 18%, la majorité de ces traumatismes était survenue par armes à feu utilisées dans un but de détournement de véhicules.^8^ Au Brésil, la plupart des TA étaient dus à des comportements déviants après une consommation excessive d’alcool ou de drogues.^9^ D’autres facteurs contribuant à alourdir la mortalité chez les traumatisés de l’abdomen rapportés sont par exemple le retard dans le diagnostic, les difficultés d’accès aux soins de qualité ou à une prise en charge chirurgicale adéquate rapide, ou encore le manque de produits sanguins labiles.^1,6,8,9^

Pays ayant le plus faible revenu dans les Caraïbes,^10^ Haïti est en proie à des violences socio-politiques récurrentes depuis quelques décennies. Depuis 2019, des pics sont observés lors de nombreuses manifestations motivées par de multiples raisons, telles les protestations anti-gouvernementales, la cherté de la vie, la pénurie de carburant, les affrontements entre gangs organisés. Port-au-Prince la capitale, est l’épicentre de ces violences, avec conséquemment une fréquence élevée de traumatismes pénétrants suite à des agressions par armes blanches, à feu ou contondants.^8^

En Décembre 2019, Médecins Sans Frontières (MSF) a porté appui au Ministère de la Santé Publique, afin d’améliorer l’accès aux soins de qualité pour la prise en charge de ces patients traumatisés. Malgré les efforts consentis, selon les données relevées au Centre de traumatologie pour le compte du deuxième semestre de 2020, la mortalité liée aux traumatismes reste toujours élevée, d’où la nécessité d’une analyse plus approfondie des réelles conditions de survenue de ces TA, ainsi que leurs caractéristiques dans ce contexte spécifique de Haïti.

Les objectifs spécifiques poursuivis au cours de cette étude réalisée dans ledit centre, entre janvier 2020 et décembre 2021 étaient de déterminer : 1) les caractéristiques sociodémographiques et cliniques des patients victimes de TA, 2) leur tendance dans le temps et les circonstances de survenue ; et 3) l’issue thérapeutique ; et 4) les principaux facteurs associés à une issue défavorable.

## MÉTHODOLOGIE

### Type d’étude

Il s’agissait d’une étude transversale sur des données rétrospectives colligées sur les traumatisés abdominaux suivis dans le centre entre janvier 2020 et ­décembre 2021.

### Cadre de l’étude

Le Centre de Traumatologie MSF-Tabarre de Port-au-Prince a été construit pour améliorer la prise en charge des urgences chirurgicales. Cet hôpital de 50 lits possède avec un service d’urgence 24/7, de soins intensifs de 6 lits, et est doté d’une banque de sang. Malgré cela, à l’instar d’autres pays à faibles revenus,^11^ le manque de produits sanguins labiles est courant ; ce qui complique la prise en charge en routine.^12^ Le centre fournit des soins gratuits pour la population.

### Population d’étude

Tous patients admis au Centre de Traumatologie MSF-Tabarre pour traumatisme entre janvier 2020 et décembre 2021 étaient inclus.

### Variables

Les principales variables collectées étaient le délai d’arrivée, les événements socio-politiques, les données socio-démographiques, les modes de survenue, le mode d’admission, la durée d’hospitalisation et le devenir des patients. Elles ont été extraites de la base de données électronique utilisée à MSF-Tabarre dans un fichier Excel (Microsoft, Redmond, WA, USA) et ont été anonymisées.

### Analyse statistique

Les données ont été analysées avec le logiciel Epi Info v7.2.4.0 (Centers for Disease Control and Prevention, Atlanta, GA, USA). Les caractéristiques de base ont été décrites en utilisant les fréquences et les pourcentages pour les variables catégorielles, les médianes et les intervalles interquartiles (IQR) pour les variables continues. Une association entre une issue défavorable (décès, référence, sortie contre-avis médical) et certains facteurs explicatifs (transfusion sanguine, délai d’arrivée, séjour à l’hôpital et évènements socio-politiques) était recherchée par régression logistique simple. Le rapport de risque (RR), son intervalle de confiance à 95% et la *P*-value étaient déterminés. Les tests de χ^2^ ou de Fisher Exact étaient utilisés. Deux médianes étaient comparées en utilisant le test de Kruskal–Wallis. Le seuil de significativité était fixé à 5%.

### Considérations éthiques

L’accord du Comité National d’Ethique et de Recherche en Haïti a été obtenu (Réf 2122-30). Cette recherche remplissait les critères d’exemption fixés par le Comité d’éthique de MSF pour les analyses a posteriori des données cliniques collectées de manière routinière et ne nécessitait donc pas d’examen MSF Ethics Review Board (Génève, La Suisse). Elle a été menée avec l’autorisation du directeur Médical du Centre Opérationnel de Paris.

## RÉSULTATS

**FIGURE 1:**
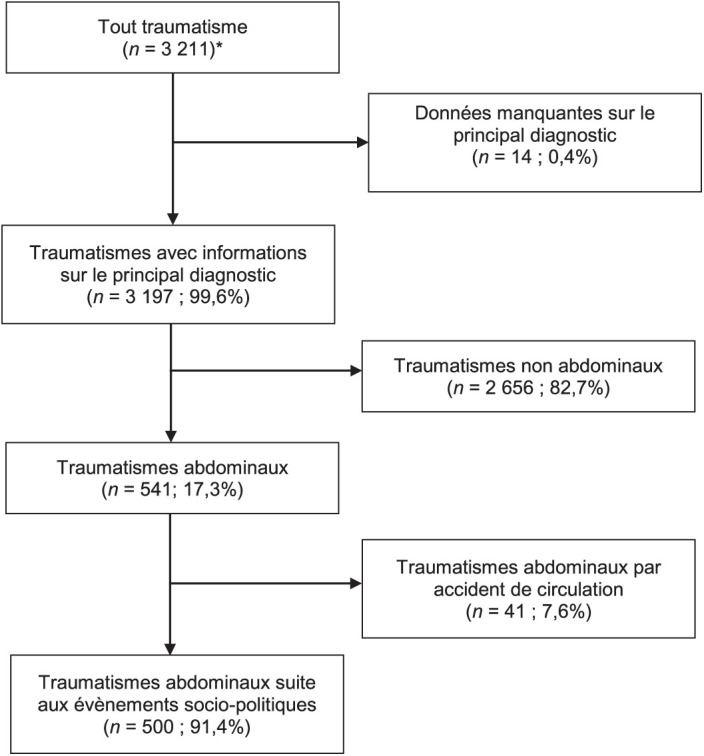
Description du processus de sélection des patients admis à MSF-Tabarre (Port-au-Prince, Haïti) pour traumatisme abdominal post-violences sociopolitiques, 2020–2021. *Pour 18 patients le décès est survenu aux urgences, dont sept décès dus au TA ; ils n’ont pas été considérés comme des admissions, et n’ont pas été inclus dans l’étude. MSF = Médecins Sans Frontières ; TA = traumatismes abdominaux.

**FIGURE 2:**
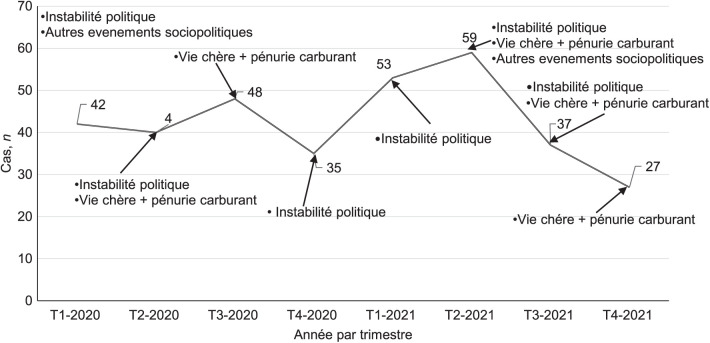
Evolution des cas de traumatisme abdominal* dans le temps et avec les événements sociopolitiques à l’origine des violences, admis au MSF-Tabarre, Port-au-Prince, Haïti, 2020–2021. *Des 500 TA, pour 341, l’évènement socio-politique était mentionné. MSF = Médecins Sans Frontières ; T = trimestre ; TA = traumatismes abdominaux.

De janvier 2020 à décembre 2021, 3 211 patients victimes de traumatismes ont été admis à MSF-Tabarre. Le principal diagnostic était renseigné chez 3 197 (99,6%). D’eux, 541 (17,3%) patients avaient un TA, dont 500 (91,4%) en lien avec des événements sociopolitiques, après avoir exclu ceux provoqués par des accidents de la circulation (*n* = 41 ; 7,6%). Ces patients représentent aussi 17,3% de l’ensemble des traumatisés admis pendant la période d’étude (Figure 1). Leur âge médian était de 30 ans (IQR 23–38) et la majorité était masculin (*n* = 429 ; 85,8%). Les TA étaient survenus dans un contexte d’instabilité politique chez 171 (34,2%), de manifestations contre la cherté de la vie ou la pénurie de carburant chez 148 (29,6%). Ils étaient occasionnés par blessure par balle chez 371 (74,2%). La distance médiane séparant le lieu de la violence de l’hôpital était de 11 km (7–15) ; et chez 231 (46,2%) la distance à parcourir était inférieure ou égale à 10 km (Tableau 1). En considérant l’ensemble des 2 326 cas de traumatismes post-violences, les TA représentaient aussi 21,5%. Parmi eux, 9 (1,8%) étaient venus dans l’heure suivant le traumatisme ; et 98 (19,6%) dans les 3 heures. La transfusion n’était pas réalisée bien qu’indiquée, ou était insuffisante chez 169 (33,8%). L’issue était défavorable chez 57 (11,4%), incluant 8,0% de décès (*n* = 40) (Tableau 2). Quant aux événements survenus dans le temps, l’instabilité politique a été le plus fréquent pendant la période d’étude, particulièrement pendant les trois premiers trimestres de 2021, avec le pic de TA le plus élevé enregistré au deuxième trimestre (*n* = 59 ; 17,3%) (Figure 2). En s’intéressant plus spécifiquement à l’issue défavorable, le risque était élevé chez les patients insuffisamment transfusés (RR 2,4 ; IC 95% 1,4–4,3 ; *P = *0,006), alors qu’il était apparu moindre chez ceux qui ne l’étaient pas du tout (RR 0,4 ; IC 95% 0,1–0,9 ; *P* = 0,027), comparés aux patients ayant eu une transfusion suffisante. De même, une blessure par balle était associée à un risque élevé d’issue défavorable (RR 2,4 ; IC 95% 1,1–5,2 ; *P* = 0,002). La durée médiane du séjour hospitalier était significativement plus courte en cas d’issue défavorable (1 jour vs. 6 jours ; *P* < 0,00) (Tableau 3)

**TABLEAU 1:** Caractéristiques sociodémographiques des patients admis pour traumatismes à MSF-Tabarre, Port-au-Prince, Haïti, 2020–2021

Caracteristiques sociodemographique et politique	Trauma non abdominal (*n* = 1 826) *n* (%)	Trauma abdominal (*n* = 500) *n* (%)	Tous traumas confondus (*n* = 3 211)* n (%)
Age, ans, médiane [IQR]	30 [23–39]	30 [23–38]	31 [24–40]
Sexe			
Masculin	1 519 (83.2)	429 (85.8)	2 638 (82.2)
Feminin	307 (16.8)	71 (14.2)	573 (17.8)
Evènement socio-politique			
Instabilité politique	550 (30.1)	171 (34.2)	989 (30.8)
Vie chère + pénurie carburant	531 (29.1)	148 (29.6)	922 (28.7)
Autres évènements politiques	26 (1.4)	32 (6.4)	58 (1.8)
Données manquantes	719 (39.4)	149 (29.8)	1 243 (38.7)
Origine du trauma			
Blessure par balle	1 381 (75.6)	371 (74.2)	1 752 (54.6)
Accident de la circulation			885 (27.6)
Blessure par arme blanche	272 (14.9)	112 (22.4)	384 (12.0)
Choc direct	3 (0.2)	5 (1.0)	8 (0.2)
Autre	169 (9.3)	12 (2.4)	181 (5.6)
Données manquantes	1 (0.1)		1 (0.0)
Proximité patient-Tabarre : distance, km, médiane [IQR]	(*n* = 1 805) 9 [7–15]	(*n* = 490) 11 [7–15]	(*n* = 3 161) 9 [7–15]
<5	145 (7.9)	38 (7.6)	237 (7.5)
5–10	836 (45.8)	193 (38.6)	1 388 (43.2)
11–15	222 (12.2)	70 (14.0)	348 (10.8)
16–20	357 (19.6)	99 (19.8)	635 (19.8)
?20	245 (13.4)	90 (18.0)	553 (17.2)
Données manquantes	21 (1.2)	10 (2.0)	50 (1.6)

*Inclue les 14 données manquantes et les 885 traumatismes dus aux accidents de la circulation. MSF = Médecins Sans Frontières ; IQR = *interquartile range* (intervalle interquartile)

**TABLEAU 2:** Caractéristiques médicales des patients avec un traumatisme admis à MSF-Tabarre, Port-au-Prince, Haïti, 2020–2021

Caractéristiques cliniques	Traumatisme abdominal (*n* = 500)* n (%)	Tous traumatismes confondus (*n* = 2 326)* n (%)
Modalité de référence		
Spontanée	237 (47.4)	1 378 (58.9)
Referee d’un autre centre	262 (52.4)	947 (41.1)
Données manquantes	1 (0.2)	1 (0.0)
Délai d’arrivée, h		
<1	9 (1.8)	16 (0.7)
1–3	89 (17.8)	180 (7.6)
4–6	198 (39.6)	358 (15.1)
7–12	56 (11.2)	101 (4.3)
>12	27 (5.4)	55 (2.3)
Données manquantes	121 (24.2)	1 616 (70.0)
Diagnostic principale		
Fracture ouverte d’un membre		711 (30.6)
Lésion superficielle		561 (24.1)
Trauma abdominal	500 (100.0)	500 (21.5)
Trauma thorax		430 (18.5)
Autre pathologie		100 (4.3)
Trauma tête		10 (0.4)
Données manquantes		14 (0.6)
Transfusion		
Non indiquée	155 (31.0)	879 (37.8)
Oui	151 (30.2)	225 (9.7)
Insuffisante	47 (9.4)	58 (2.5)
Non reçue	122 (24.4)	670 (28.8)
Données manquantes	25 (5.0)	494 (21.2)
Durée de séjour dans l’USI, jours, médiane [IQR]	(*n* = 193) 4 [2–6]	(*n* = 316) 3 [2–6]
Devenir^†^		
Décédé	40 (8.0)	57 (2.5)
Guéri	354 (70.8)	1 547 (66.5)
Parti sans autorisation	12 (2.4)	30 (1.3)
Référé	5 (1.0)	134 (5.8)
Données manquantes	34 (6.8)	558 (24.0)
Durée de séjour à l’hôpital, jours, médiane [IQR]	(*n* = 456) 6 [2–9]	(*n* = 1 665) 2 [0–6]

*Tout traumatisme post-violences sociopolitiques.

^†^Issue défavorable = référé + décès + parti sans autorisation.

MSF = Médecins Sans Frontières ; IQR = *interquartile range* (intervalle interquartile) ; USI = unités de soins intensifs.

**TABLEAU 3:** Facteurs à l’issue favorable associés à l’issue défavorable chez des patients admis pour traumatisme abdominal post-violences socio-politiques à MSF-Tabarre, Port-au-Prince, Haïti, 2020–2021

Facteurs	Issue défavorable* n (%)	Issue favorable^†^ *n* (%)	*P*-value (χ^2^/Fisher Exact)	RR (IC 95%)
Evènements socio-politiques			0.839	
Instabilité politique	17 (44.7)	149 (50.2)	0.702	0.8 (0.3–2.3)
Vie chère + pénurie carburant	17 (44.7)	120 (40.4)	0.988	1.0 (0.4–2.8)
Autres	4 (10.5)	28 (10.5)		Référence
Délai d’arrivée, h			0.690	
0–3	13 (29.5)	85 (25.7)		Référence
4–6	20 (45.5)	174 (52.7)	0.451	0.8 (0.4–1.5)
7–12	7 (15.9)	48 (14.5)	0.700	0.8 (0.3–2.1)
>12	4 (9.1)	23 (7.0)	0.100	1.1 (0.4–3.2)
Transfusion			<0.000	
Faite et suffisante	21 (51.2)	129 (49.0)		Référence
Faite et insuffisante	16 (37.2)	31 (11.8)	0.006	2.4 (1.4–4.3)
Non faite	5 (11.6)	103 (39.2)	0.027	0.4 (0.1–0.9)
Origine de trauma				
Arme blanche + autres	7 (12.3)	111 (27.1)		Référence
Blessure par balle	50 (87.7)	298 (72.9)	0.002	2.4 (1.1–5.2)
Distance de Tabarre, km, médiane [IQR]	9 [7–15]	11 [7–15]	0.14^‡^	
Durée de séjour à l’hôpital, jours, médiane [IQR]	1 [0–3]	6 [3–10]	0.00^‡^	

*Issue défavorable : décédé, référé, parti sans autorisation.

^†^Issue favorable : guéri, sortie avec suivi médical.

^‡^Kruskal–Wallis.

MSF = Médecins Sans Frontières ; IQR = *interquartile range* (intervalle interquartile) ; RR = rapport de risque ; IC = intervalle de confiance.

## DISCUSSION

De ce travail, nous retenons que les TA sont survenus près de deux patients sur 10 admis pour traumatisme ; et que d’eux, l’issue était défavorable chez un patient sur 10, le plus souvent par décès. En Haïti, les TA sont de loin causés par les violences sociopolitiques, dans neuf cas sur 10. Les principales raisons de ces mouvements d’humeur étaient l’instabilité politique et les protestations contre les conditions de vie. Mieux l’accumulation des dites causes pendant la même période était corrélée à l’augmentation de ces cas. Les personnes les plus impliquées étaient des sujets adultes jeunes, âgés de 30 ans. En Haïti, ce sont plus souvent des jeunes hommes qui vont manifester et qui sont le plus souvent muni d’armes que les femmes. Cela semble expliquer pourquoi notre cohorte de patients était principalement composée de jeunes hommes. Ce qui est comparable à d’autres études où la violence est le principal instigateur des traumatismes abdominaux comme décrit par Ogbuanya et al.^13^ Les lésions abdominales faisaient suite à des blessures par balle chez près des trois quarts d’entre eux, avec une issue plus défavorable démontrée. Ces résultats corroboraient ceux d’autres auteurs dans la littérature.^1^ Même si un cas clinique rapporté en Haïti n’a pas retrouvé de dommages après TA par arme à feu, après laparotomie,^14^ ce fait est certainement exceptionnel.

Après blessure, la distance séparant le lieu de violences de l’hôpital ne dépassait pas 10 km chez la moitié des patients ; et pourtant, seulement une personne sur cinq réussissait à se rendre à l’hôpital dans l’intervalle des 3 heures. Le recours rapide à des soins de qualité est un élément essentiel à la survie des traumatisés ; les conséquences néfastes de tout retard dans la prise en charge de tels patients, particulièrement dans les pays aux ressources limitées, ont déjà été relevées par d’autres auteurs.^5,8,15^ Les causes en sont multiples, incluant le manque d’équipements, d’ambulances, la mauvaise qualité des infrastructures routières, mais aussi la distance à parcourir.^6^ Cette situation est loin d’être spécifique aux traumatisés puisqu’une étude multicentrique à laquelle avait participé Haïti avait déjà relevé un long délai d’arrivée aux urgences pour les femmes et les enfants non-traumatisés.^12^ Le contraste observé entre la faible distance à parcourir et le long délai mis pour parvenir à l’hôpital s’explique dans notre contexte par l’insécurité permanente qui limite le déplacement des ambulances, situation aggravée pendant la nuit.

Une fois en milieu hospitalier, la prise en charge de ces patients fait appel entre autres à la transfusion, puisque la majorité des décès évitables en traumatologie est liée à des hémorragies graves.^12^ Un précédent travail mené en Haïti avait rapporté la fréquence d’hémorragies non négligeables et la nécessité de transfuser pour éviter le décès chez les traumatisés abdominaux.^15^ Cette transfusion n’a pas été faite ou était insuffisante chez le tiers des patients. La transfusion sanguine constitue une préoccupation quotidienne en Haïti, où le contexte sociopolitique limite voire empêche la collecte de sang, provoquant ainsi une inadéquation entre l’offre et la demande. Logiquement, les transfusions insuffisantes étaient associées à une issue défavorable, du fait de la mise en jeu habituelle du pronostic vital. Par contre, nous avons été surpris par l’association significative ­entre l’absence de transfusion et une issue plutôt favorable, contrairement aux données scientifiques. Une tentative d’explication pourrait être la priorisation des cas par les médecins selon la gravité de l’état clinique, attitude nécessaire devant la pénurie de produits sanguins labiles. Ainsi, les cas moins graves qui n’auraient pas bénéficié d’une transfusion immédiate, se seraient rapidement améliorés, grâce à l’arrêt du saignement et à une stimulation de l’érythropoïèse. Dans tous les cas, cette situation nécessite mûres réflexions, voire des investigations supplémentaires.

Les patients qui ont eu un séjour hospitalier plus court (médiane : 1 jour) ont eu significativement une issue défavorable que ceux qui ont eu un séjour plus long (médiane : 6 jours), ce qui est différent d’une étude tanzanienne où, un séjour hospitalier de ≥7 jours augmentait le risque de mortalité.^16^ Dans cette étude, les critères d’inclusion étaient différents, n’incluant que les patients ayant bénéficié d’une intervention et la période de suivi, alors que dans notre étude, certains patients sont décédés le jour même de leur arrivée, alors qu’ils avaient déjà été admis, ou dans les jours qui suivent.

### Limites et forces de l’étude

Bien que plusieurs études aient été précédemment menées en Haïti sur les traumatismes, la singularité de celle-ci est d’avoir essayé d’analyser les TA et leur tendance évolutive dans le temps, à la lumière des événements socio-politiques ayant sévi dans le pays. De plus, plusieurs sources de données relatives aux patients admis pour TA ont été utilisés afin de recueillir le plus d’informations possibles. Par ailleurs, les autres forces de l’étude résident dans le nombre assez élevé de patients inclus et dans le fait d’avoir considéré tous ceux qui étaient admis pendant la période, permettant ainsi de limiter les biais liés aux fluctuations d’échantillonnage. Toutefois, l’étude comporte aussi certaines limites. Etant de nature rétrospective, seules les informations disponibles dans la base de données de l’hôpital ont été exploitées. De même, des précisions sur certaines variables telles que les évènements socio-politiques, le délai d’arrivée et la transfusion sanguine manquaient. Bien qu’elles aient été complétées autant que possible à partir des registres et des dossiers personnels des patients, il en manquait encore un certain nombre, ce qui aurait pu fausser nos conclusions. Enfin, il était difficile de colliger certaines variables importantes comme les taux d’hémoglobine, qui auraient pu contribuer à réévaluer l’indication d’une transfusion après des jours d’hospitalisation chez les patients qui n’étaient pas immédiatement transfusés.

### Implications opérationnelles

Ce travail donne l’opportunité de plaider auprès des forces gouvernementales du pays pour la mise en place de mesures pour un recours rapide aux soins, afin d’améliorer le pronostic des patients. Elles pourraient passer par une meilleure sécurisation des ambulances et des traumatisés sur le trajet menant vers l’hôpital. De même, il importe de mettre en place des stratégies innovantes pour l’approvisionnement ininterrompu en produits sanguins labiles, même en période de crise.

## CONCLUSION

En conclusion, pendant les évènements socio-politiques survenus entre 2020 et 2021, plusieurs cas de TA souvent par balle ont été admis au service d’urgence de MSF-Tabarre, mais dans des délais relativement trop longs pour de courtes distances. La pénurie de produits sanguins a eu un impact sur les résultats des patients. Des efforts sont nécessaires pour un recours rapide aux soins en cas de traumatisme et une disponibilité permanente en produits sanguins labiles.

### Remerciements

Cette recherche a été menée dans le cadre de l’Initiative de recherche opérationnelle et de formation structurée (SORT-IT), un partenariat mondial dirigé par le Programme spécial de recherche et de formation sur les maladies tropicales de l’Organisation mondiale de la santé (OMS/TDR). Le modèle est basé sur un cours élaboré conjointement par l’Union internationale contre la tuberculose et les maladies respiratoires (L’Union) et Médecins Sans Frontières. Le programme spécifique, SORT-IT, qui a donné lieu à cette publication a été organisé par MSF spécifiquement pour la recherche en langue français.

Le programme a été financé par La Fondation Veuve Emile Metz-Tesch, Luxembourg. Le financeur n’a joué aucun rôle dans la conception de l’étude, la collecte et l’analyse des données, la décision de publier ou la préparation du manuscrit.
